# Panoramic Image Driven Point Cloud Initialization for 3D Reconstruction

**DOI:** 10.3390/s25226840

**Published:** 2025-11-08

**Authors:** Haoyu Qian, Lidong Yang, Jing Wang, Muhammad Shahid Anwar

**Affiliations:** 1 School of Digital and Intelligent Industry, Inner Mongolia University of Science and Technology, Baotou 014010, China; hyw5638@gmail.com; 2 Inner Mongolia Key Laboratory of Pattern Recognition and Intelligent Image Processing, Baotou 014010, China; 3 School of Information and Electronics, Beijing Institute of Technology, Beijing 100811, China; wangjing@bit.edu.cn; 4 Interdisciplinary Research Center for Finance and Digital Economy, King Fahd University of Petroleum and Minerals, Dhahran 31261, Saudi Arabia; muhammad.anwar.1@kfupm.edu.sa

**Keywords:** 3D reconstruction, panoramic image, point cloud initialization

## Abstract

The ability to reconstruct immersive and realistic three-dimensional scenes plays a fundamental role in advancing virtual reality, digital twins, and related fields. With the rapid development of differentiable rendering frameworks, the reconstruction quality of static scenes has been significantly improved. However, we observe that the challenge of insufficient initialization has been largely overlooked in existing studies, while at the same time heavily relying on dense multi-view imagery that is difficult to obtain. To address these challenges, we propose a pipeline for text driven 3D scene generation, which employs panoramic images as an intermediate representation and integrates with 3D Gaussian Splatting to enhance reconstruction quality and efficiency. Our method introduces an improved point cloud initialization using Fibonacci lattice sampling of panoramic images, combined with a dense perspective pseudo label strategy for teacher–student distillation supervision, enabling more accurate scene geometry and robust feature learning without requiring explicit multi-view ground truth. Extensive experiments validate the effectiveness of our method, consistently outperforming state-of-the-art methods across standard reconstruction metrics.

## 1. Introduction

With the widespread adoption of virtual reality (VR) devices and content, immersive and realistic 3D reconstructions have become increasingly important. Traditional scanning pipelines can capture accurate geometric structures and spatial relationships but are costly in both time and labor. Recent developments in deep learning have given rise to learning-based reconstruction approaches such as photogrammetry [1,2,3,4], Neural Radiance Fields (NeRFs) [5,6,7,8], and 3D Gaussian Splatting (3DGS) [9,10,11,12,13,14]. Although these methods have achieved impressive results, they typically depend on multi-view observations to build complete spatial understanding, requiring high quality imaging with multiple or moving cameras, posing a barrier for general users.

Reliable depth estimation is the cornerstone of these reconstruction pipelines because it links image data to 3D geometry. Structure-from-motion (SfM) and multi-view stereo (MVS) rely on feature matching and photometric consistency to estimate dense disparity across views. Despite numerous improvements from learning-based approaches [15,16,17], depth prediction remains unreliable under sparse views, small baselines, or weak textures. Several studies on sparse view depth estimation [18,19,20] attempt to predict depth from limited inputs, but reconstruction quality remains constrained by the lack of parallax and multi-view consistency.

To mitigate dependence on multi-view capture, we adopt panoramas as alternative sources of global scene context. A single panorama provides a comprehensive view of an entire scene within one frame, offering broader spatial coverage and richer contextual cues than conventional perspective images. Recent latent diffusion models, such as MVDiffusion [21] and StitchDiffusion [22], have demonstrated strong capability in generating high fidelity panoramic images from textual descriptions, enabling users to obtain realistic scene priors directly from text. These developments suggest that panoramic representations can potentially replace multi-view inputs for reconstruction by providing rich global context.

Although panoramas provide global coverage, they lack parallax, making depth estimation inherently ambiguous and unstable. Monocular and panoramic depth estimation methods [23,24] partially alleviate this issue, yet they remain susceptible to scale ambiguity and instability in texture-less regions. Within 3DGS, the quality of point cloud initialization (which depends on depth accuracy) strongly affects optimization stability and final reconstruction quality. Existing pipelines often initialize 3DGS using SFM, such as COLMAP [25] and FlowMap [26]. While these methods recover camera poses and sparse geometry effectively, their performance degrades when texture information is limited. Thus, a robust, panorama-aware initialization is essential for preserving fine Gaussian details and ensuring stable optimization.

To this end, we propose a novel pipeline that generates a 3D Gaussian representation specifically designed for panoramic formats. The method leverages text-to-panorama generation to efficiently recover comprehensive 3D scenes from a single panoramic image, enhancing reconstruction quality by exploiting the wide field of view and rich contextual cues of panoramic imagery. Advanced text generation techniques are first applied to refine prompt quality and image boundaries through adaptive weight smoothing. Multiple overlapping perspective projections are then generated as pseudo labels, which transfer feature knowledge to a student model via knowledge distillation. During initialization, Fibonacci sampling determines camera positions and intrinsic parameters to produce multi-view depth maps, which are transformed into spherical coordinates and logarithmically compressed to reduce depth variation. The optimized depth is remapped into 3D space to form a detailed point cloud, which is subsequently converted into optimizable 3D Gaussian primitives. The continuous Gaussian representation fills depth gaps, while transmittance weighting distinguishes surface from internal Gaussians, preserving crucial structural details.

In summary, our work introduces the following key contributions:

- We propose a novel 3D Gaussian representation pipeline that leverages text-to-panorama generation to enhance scene reconstruction performance.
- Our method employs Fibonacci sampling for point cloud initialization, which substantially improves reconstruction quality.
- Our method employs dense pseudo labels to guide student model learning from the teacher model, and results show consistent improvements over deterministic teachers.
- Extensive experiments demonstrate that our method achieves higher quality reconstructions than SOTA approaches.

## 2. Methods

**Figure 1:**
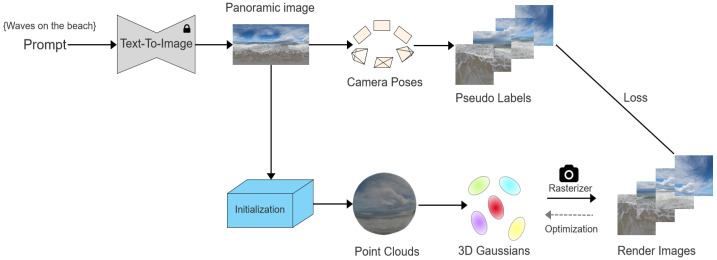
Overview of the proposed experimental framework, including text-to-panorama generation, pseudo label extraction, point cloud initialization, and adaptive Gaussian optimization.

As illustrated in Figure 1, the proposed framework begins with text-to-panorama generation using a diffusion model. The resulting panoramic images are used for pseudo label generation and point cloud initialization, which are further optimized into 3D Gaussian primitives through differentiable rasterization under loss constraints. This process integrates generative and optimization-based components for high fidelity panoramic reconstruction. Section 2.1 introduces the basic concept of 3DGS, Section 2.2 describes the text-to-panorama generation process, Section 2.3 presents dense pseudo label generation, Section 2.4 details point cloud initialization, and Section 2.5 discusses adaptive Gaussian optimization.

### 2.1. Preliminary

3DGS employs Gaussian ellipsoids [27] to effectively parallelize the representation of the scene or object intended for reconstruction. Each Gaussian contains center position μ, covariance matrix ∑, opacity α, and *SH* coefficients, collectively known as the Gaussian mean:

(1) $$G\left(x\right)=e^{-\frac{1}{2}{\left(x\right)}^{T}\sum^{-1}\left(x\right)}$$

In order to guarantee the positive semi-definiteness of the covariance matrix, it can be factorized into a scaling matrix S and a rotation matrix R. We retain the diagonal vector of the scaling matrix S and a quaternion vector of the rotation matrix R for Gaussian distributions:

(2) $$\sum =RSS^{T}R^{T}$$

During rendering, 3D Gaussians are projected onto a 2D plane, forming ellipses that are approximated as circles by maintaining their center coordinates and radius. This process entails converting a 3D covariance matrix to a 2D covariance matrix using a combination of the projective transformation matrix and the viewing transformation matrix:

(3) $$\sum^{\prime }=JW\sum W^{T}J^{T}$$

The 2D plane is partitioned into distinct blocks, with individual Gaussians associated with specific blocks, influencing all pixels within them. When there is overlap between 2D Gaussians, determining the relative depths of each Gaussian is necessary to address occlusion issues from closer Gaussians. Rendering of blocks and pixels occurs independently and simultaneously following a predefined sequence, culminating in color computation via alpha blending:

(4) $$C=\sum c_{i}\alpha_{i}^{\prime }\prod_{j=1}^{i-1}\left(1-\alpha_{j}^{\prime }\right)$$

$c_{i}$ represents the learned color, while the final opacity $\alpha_{i}^{\prime }$ is the product of the learned opacity $\alpha_{i}$ and the Gaussian function:

(5) $$\alpha_{i}^{\prime }=\alpha_{i}\times \exp\left(-\frac{1}{2}{\left(x^{\prime }-\mu_{i}^{\prime }\right)}^{T}\sum_{i}^{\prime -1}\left(x^{\prime }-\mu_{i}^{\prime }\right)\right)$$

where $x^{\prime }$ and $\mu_{i}^{\prime }$ represent the coordinates in the projection space.

### 2.2. Text to Panoramic Image

**Figure 2:**
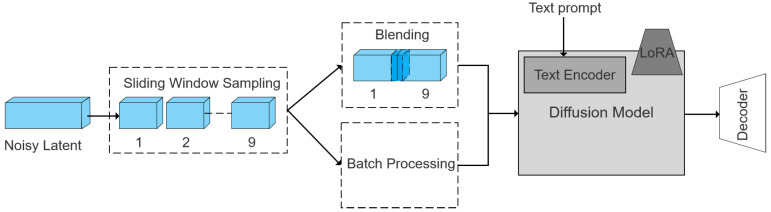
Pipeline of latent panoramic image generation using sliding window sampling and adaptive blending within the diffusion denoising process.

We employ a latent denoising strategy that combines sliding window sampling with adaptive weight blending to reduce discontinuities at the left and right boundaries; the entire procedure is detailed in Figure 2. Initially, the noisy latent $z_{t}$ is divided into nine overlapping patches $P_{i}(z_{t})$ through sliding window sampling. Next, the text prompt is encoded with a pretrained text encoder, optionally augmented with a LoRA [28] module for refined parameter adjustment. The resultant text embedding is then integrated into the diffusion model Φ at each denoising step, influencing the denoising process of every latent patch; this process can be mathematically expressed as:

(6) $$\Phi \left(z_{t-1}\right)=\sum_{i=1}^{n}\frac{P_{i}^{-1}\left(1\right)}{\sum_{j=1}^{n}P_{j}^{-1}\left(1\right)}⨂P_{i}^{-1}(\Phi (P_{i}(z_{t})))$$

Additionally, an adaptive weight w is employed to blend the initial left and final right patches. Subsequently, denoising is also applied to the blended patch to maintain coherence in the border areas, the formula for this process is expressed as:

(7) $$blended=w*left+\left(1-w\right)*right$$

After integrating and weighting the outputs of the diffusion model, the resulting latent representation is passed to the decoder for generation. To ensure high fidelity, the central region is retained in the ultimate output.

### 2.3. Dense Perspective Pseudo Label Generation for Teacher–Student Distillation Supervision

The inherent inconsistency among Gaussian distributions requires individual optimization of their properties using image supervision. However, panoramic images inherently lack explicit multi-view information, limiting their effectiveness in tasks demanding precise geometric comprehension and reconstruction. To overcome this limitation, we introduce a framework for creating dense perspective pseudo labels to facilitate teacher–student distillation supervision.

Given the panoramic image *P* and a designated camera position, we project it onto *N* overlapping perspective tangent images. Prior studies suggest that using 20 tangent images sufficiently covers the spherical surface resulting from icosahedral projection. By positioning multiple sets of densely distributed virtual perspective cameras within the spherical domain and applying the same projection method, we generate dense pseudo labels that capture fine scene details.

To enhance reconstruction, we leverage a powerful pretrained model (Moge2) as the teacher model and implement knowledge distillation to transfer the acquired knowledge from the teacher model to our student model without the need for actual 3D ground truth data. Simultaneously, it functions as a supervised label to guide the acquisition of crucial feature information by the student model from the teacher model, and it enables optimization through an appropriate loss function. During the training phase, the loss function is defined as the following weighted sum:

(8) $$L_{total}=\left(1-\lambda_{DSSIM}\right)L_{L1}+\lambda_{DSSIM}L_{SSIM}+\omega_{depth}L_{Pearson}$$

Specifically:

(9) $$\;\;L_{L1}=\frac{1}{N}\sum_{i=1}^{N}\left|y_{i}-\hat{y_{i}}\right|$$

where $y_{i}$ is the true value, $\hat{y_{i}}$ is the predicted value, and N is the total number of elements.

(10) $$L_{Pearson}=1-\rho \left(y,\hat{y}\right)$$

where ρ is the Pearson correlation coefficient.

(11) $$\rho \left(y,\hat{y}\right)=\frac{Cov\left(y,\hat{y}\right)}{\sigma_{y}\sigma_{\hat{y}}}$$

where $Cov(y,\hat{y})$ is the covariance of y and $\hat{y}$, and $\sigma_{y}$ and $\sigma_{\hat{y}}$ are the standard deviations of y and $\hat{y}$. The formula for $L_{SSIM}$ is as follows:

(12) $$L_{SSIM}=1-\frac{\left(2{\mu_{y}}^{2}{\mu_{\hat{y}}}^{2}+C_{1}\right)\left(2\sigma_{y\hat{y}}+C_{2}\right)}{\left({\mu_{y}}^{2}+{\mu_{\hat{y}}}^{2}+C_{1}\right)\left({\sigma_{y}}^{2}+{\sigma_{\hat{y}}}^{2}+C_{2}\right)}$$

With $\mu_{y}$ and $\mu_{\hat{y}}$ denoting the means, $\sigma_{y}$ and $\sigma_{\hat{y}}$ the variances, $\sigma_{y\hat{y}}$ the covariance, and $C_{1}$ and $C_{2}$ are small constants to stabilize the division.

Intuitively, $L_{L1}$ prioritizes pixel-wise reconstruction accuracy, $L_{SSIM}$ emphasizes structural similarity, and $L_{Pearson}$ enforces the consistency correlation between the predicted and supervised image depth maps. Collectively, these components facilitate the generation of reconstructed views that closely mirror the target images in terms of both visual appearance and geometric fidelity.

### 2.4. Point Cloud Construction

Improved initialization of point clouds leads to more accurate scene geometry and reduces local overfitting during the reconstruction process. We propose a Fibonacci lattice arrangement [29] to obtain crucial information from the panoramic image.

Fibonacci camera placement. Let the golden angle be $\varphi =\pi \left(3-\sqrt{5}\right)$. For N views and index, $i\in \left\{0,\ldots ,N-1\right\}$.

(13) $$\;y_{i}=-1+2\cdot \frac{i}{N},\;\;\theta_{i}=\varphi \cdot i,\;\;r_{i}=\sqrt{1-{y_{i}}^{2}},d_{i}=(r_{i}\cdot \cos{(\theta }_{i}),y_{i},r_{i}\cdot \sin\left(\theta_{i}\right))$$

Uniformity justification. The “latitude” coordinates are chosen as midpoints of N equal subdivisions in [−1, 1], so such latitude band covers the same surface area 4π/N. The azimuths follow the golden angle progression $\theta_{i}=\varphi \cdot i$, which is known to be equidistributed on the circle. Combining equal area bands with azimuthal equidistributional yields asymptotic uniformity on the sphere.

Comparative rationale. Under the same N, latitude–longitude grids suffer polar crowding; icosahedral, geodesic meshes include pentagonal defects and mild anisotropy; and spherical Poisson disk requires iterative generation. In contrast, the spherical Fibonacci set combines equal area behavior, low discrepancy, and an analytic mapping from index to direction, offering a simple and robust initializer.

Camera intrinsics and calibration matrix. The internal reference matrix K calculation method is based on the field of view angle:

(14) $$K_{i}=\left[\begin{array}{ccc} f_{x} & 0 & c_{x}\\ 0 & f_{y} & c_{y}\\ 0 & 0 & 0 \end{array}\right]$$

The focal lengths $f_{x}$ and $f_{y}$ are calculated based on the horizontal and vertical fields of view ${fov}_{x}$ and ${fov}_{y}$:

(15) $$f_{x}=\frac{w}{2\tan\left(\frac{{fov}_{x}}{2}\right)},f_{y}=\frac{h}{2\tan\left(\frac{{fov}_{y}}{2}\right)}$$

Panoramic mapping and point cloud formation. After initializing camera directions via $\left(13\right)$, spherical UV coordinates are computed for each viewpoint by transforming the depth maps into a spherical coordinate system, through a direction to spherical coordinate conversion. To ensure smooth transitions between adjacent perspectives, a logarithmic transformation is applied to compress depth value variations. Subsequently, gradients of the logarithmic depth map are determined horizontally and vertically, denoted as ${depth}_{i}$ representing the depth map from the i-th camera:

(16) $$G_{x}=\frac{\partial }{\partial_{x}}\log{(depth}_{i}),G_{y}=\frac{\partial }{\partial_{y}}\log{(depth}_{i})$$

To further enhance the smoothness, Laplacian regularization is applied, penalizing abrupt depth discontinuities:

(17) $$\nabla^{2}\log({depth}_{i})=\frac{\partial }{\partial_{x}}\log{(depth}_{i})+\frac{\partial }{\partial_{y}}\log{(depth}_{i})$$

**Figure 3:**
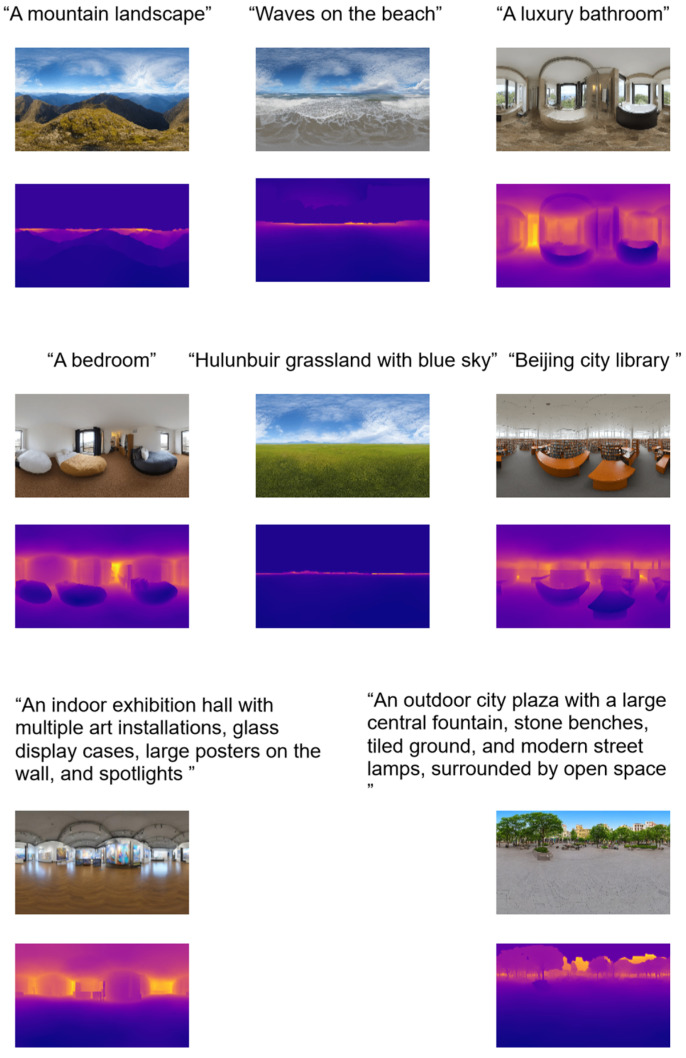
Text to panoramic image generation and corresponding depth maps. The top row shows the generated panoramic images, and bottom row displays the corresponding depth maps.

The refined panoramic depth map is then obtained by solving a least squares optimization that minimizes projection errors across multiple viewpoints while incorporating gradient and Laplacian regularization. The optimized log depth is restored via an inverse logarithmic transformation, yielding the panoramic depth maps corresponding to the generated panoramic images. Finally, the reconstructed depth values are projected into 3D space using spherical UV coordinates, yielding a detailed point cloud representation of the scene, as shown in Figure 3.

### 2.5. Adaptive Gaussian Optimization

After generating the point cloud, the Gaussian Splatting points’ centers are initialized from the input point cloud, and their position and volume are refined using supervised image guidance. Prior research on reconstructing geometry from 3D Gaussian distributions has primarily relied on strong geometric regularization, typically using opacity as a criterion and discarding Gaussians with very low opacity. However, Gaussians with low contributions often exhibit relatively high opacity, making the default strategy inadequate to maintain geometry precision, particularly under the dense initial distributions. We identify Gaussians for pruning by determining their overall contribution as the average across several high contributing viewpoints. Surface Gaussians are further distinguished from internal ones by their transmittance properties, ensuring that surface-attached Gaussians are preserved even under low transmittance conditions. Training begins with a relaxation stage aimed at reducing pruning severity, which is gradually increased over time. This strategy retains a larger number of Gaussians during the early stages to support optimization, while progressively removing redundancies in later stages.

## 3. Experiments

### 3.1. Implementation Details

In accordance with the proposed pipeline, a dataset comprising eight textual prompts was constructed to comprehensively evaluate the performance of the proposed method across diverse indoor and outdoor scenarios. The prompts included six standard descriptions: “A mountain landscape”, “Waves on the beach”, “A luxury bathroom”, “A bedroom”, “Hulunbuir grassland with blue sky”, and “Beijing city library”. In addition, two more complex scenes were introduced to examine spatial complexity and geometric generalization, namely, “An indoor exhibition hall with multiple art installations, glass display cases, large posters on the wall, and spotlights” and, “An outdoor city plaza with a large central fountain, stone benches, tiled ground, and modern street lamps surrounded by open space.”

All experiments were conducted using Pytorch 2.4.0 with CUDA 12.4 on a workstation equipped with an NVIDIA RTX 3090 GPU (24 GB memory). Each training session consisted of 10,000 iterations to ensure stable convergence and consistent reconstruction performance.

To comprehensively evaluate our method, we employed three established reconstruction metrics: PSNR, SSIM, and LPIPS. These metrics collectively gauge pixel level accuracy, structural fidelity, and perceptual realism. Leveraging the dense pseudo labels that encompass the entire panoramic scene, we directly compared the reconstructed outputs with these pseudo labels to provide an objective and dependable assessment.

### 3.2. Comparison with Baselines

Baselines. We compared our method with three representative approaches to text-to-3D generation and panoramic scene reconstruction. LucidDreamer [30] iteratively enhances a single image and its textual prompt to generate multi-view consistent content, progressively expanding the scene to form a holistic view. To ensure fair comparison, we adapted its pipeline to accept an initial panoramic image as input and integrated our dense pseudo label supervision into its training. DreamScene360 [31] constructs immersive 360° panoramic scenes from textual prompts by projecting generated images into 3D environments. While it preserves global scene coherence, the projection process often leads to local geometric distortions, especially near high curvature regions. Scene4U [32] introduces a panoramic image-driven framework for immersive 3D scene reconstruction that enhances scene integrity by removing distracting elements. The method generates panoramic with specific spatiotemporal attributions, decomposes them into semantic layers, and refines each layer through inpainting and depth restoration before reconstructing a multi-layered 3D scene using 3DGS. Since the official implement is unavailable, we reproduced a variant following its multi-layer decomposition principle to ensure consistency within our framework.

**Figure 4:**
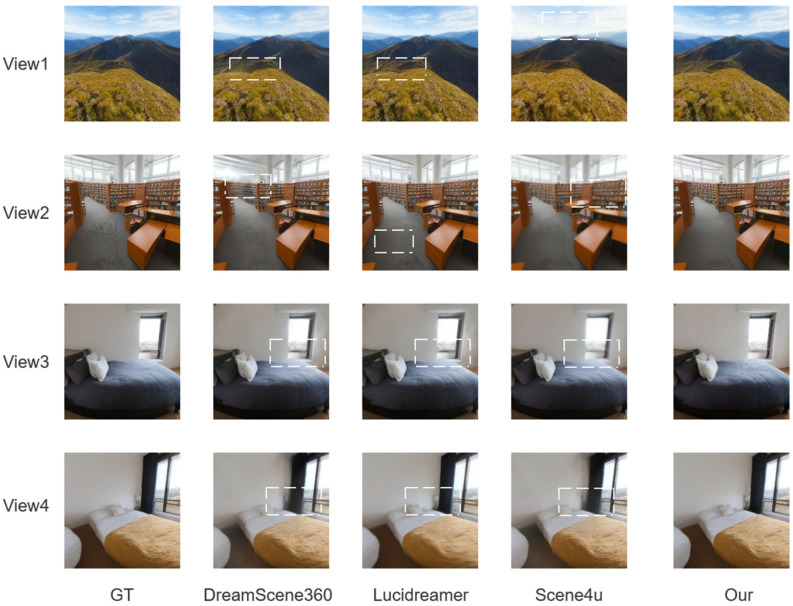
Representative examples, randomly selected from the test set, are shown for visual comparison. The white dashed boxes highlight the differences. The reconstruction quality of our method demonstrates superior recovery of both geometry and texture details.

Qualitative results. Figure 4 presents visual comparisons with baseline methods. Our method exhibits sharper textures, cleaner structural boundaries, and fewer rendering artifacts. In outdoor scenes, fine grained details in vegetation and terrain are preserved while maintaining global structural consistency. In indoor, object contours and furniture edges are preserved without the blurring and blocky distortions seen in baseline outputs.

**Figure 5:**
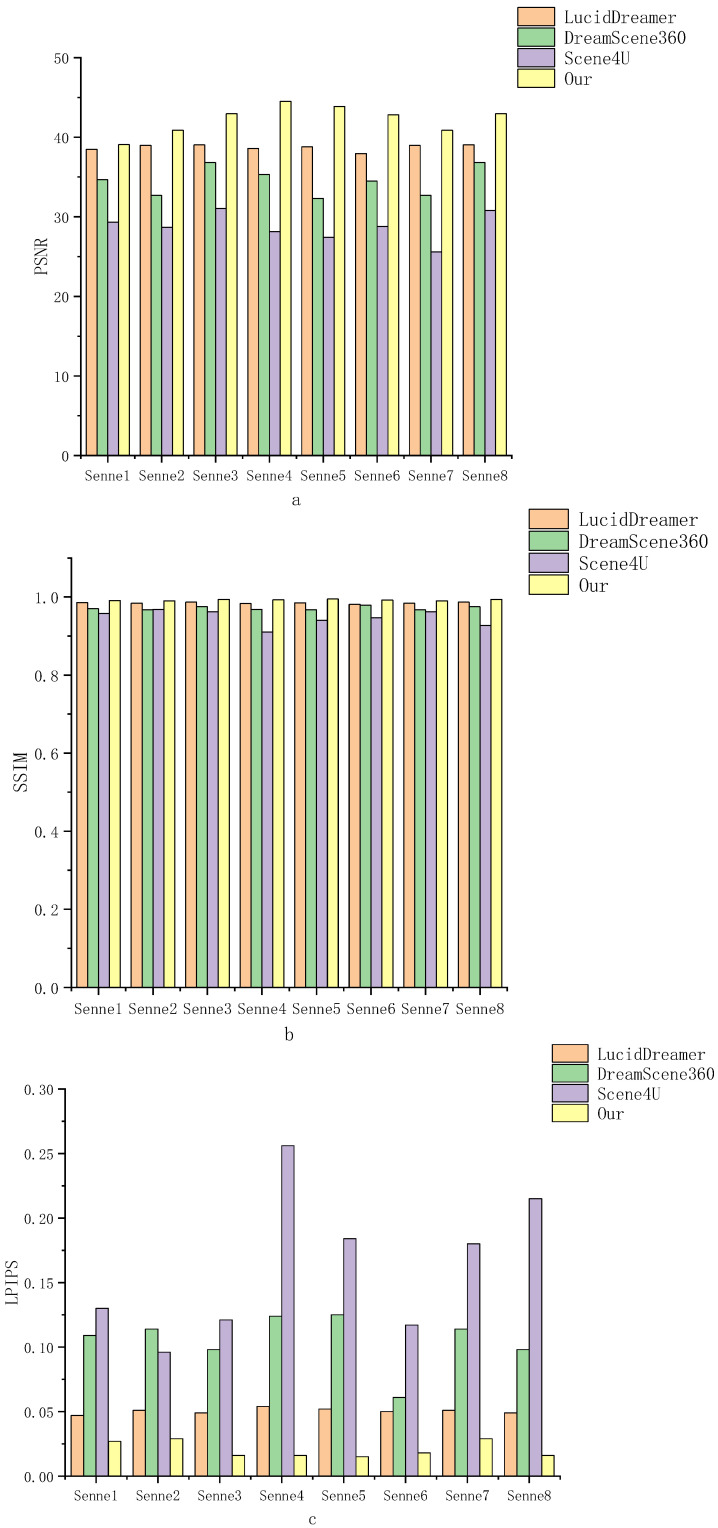
Quantitative comparison, (**a**) PSNR, (**b**) SSIM, (**c**) LPIPS. Results are reported on eight scenes indexed as Scene1–Scene8.

Quantitative results. Figure 5 summarizes performance across all eight datasets in (a) PSNR, (b) SSIM, and (c) LPIPS, and scenes are indexed as Scene1–SceneN; the mapping to full scene name is provided in Table 1. In outdoor scenes like “Hulunbuir grassland with blue sky”, our method achieves over a 5 dB improvement in PSNR score. In indoor scenarios such as “A bedroom”, our method achieves the highest SSIM and lowest LPIPS, indicating better structural similarity and perceptual realism. We attribute these gains to the improved rendering capabilities of Gaussian Splatting and our initialization strategy, which seeds a higher, more uniformly distributed set of points, enabling accurate recovery of critical details throughout the scene.

### 3.3. Ablation Study and Analysis

Ablation on point cloud initialization. We conducted ablation studies to evaluate the effectiveness of our point cloud initialization scheme against several mainstream approaches, including BiFuse, Depth anything V2 [33], VGGT [34], COLMAP, COLMAP (MVS), and FlowMap. Specifically, BiFuse fuses ERP and CubeMap projections through a dual-branch network; Depth anything V2 leverages large-scale pseudo labeled data for robust monocular depth estimation; VGGT introduces a geometry transformer capable of directly predicting depth and point clouds; COLMAP and COLMAP (MVS) provide classical sparse and dense reconstructions; and FlowMap jointly optimizes depth and camera parameters in a differentiable framework. As summarized in Table 2, our method achieves the best overall performance across all three metrics, with average scores of 42.07, 0.992, and 0.020, respectively.

**Figure 6:**
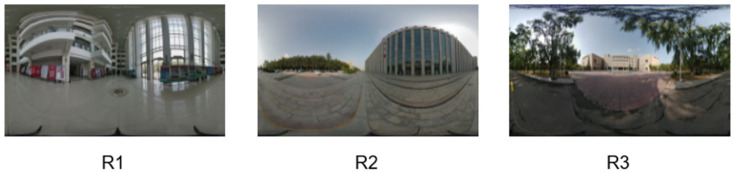
Real world panoramic images captured by the Teche 360 camera: (**R1**) indoor exhibition hall, (**R2**) laboratory exterior, (**R3**) public park.

Generalization experiments on real world panoramic data. To further assess the generalization capability and robustness of the proposed approach in real world environments, a supplementary dataset was collected using a Teche 360 panoramic camera (Teche, Shenyang, China). The first scene was an indoor exhibition hall on the first floor, the second scene was the exterior area of the laboratory building, and the third scene was a public park adjacent to the university library, as illustrated in Figure 6. For each of the three scenes, we conducted comparative experiments to quantitatively assess reconstruction performance. The detailed numerical results are summarized in Table 3, where our method consistently outperforms other methods.

Teacher model comparison. To validate the rationality of selecting Moge2 as the teacher model in our distillation framework, we conducted comparative experiments using three alternative teachers: DPT, Metrics3D, and VGGT. All training settings and evaluation metrics were kept identical to ensure fairness. As summarized in Table 4, our method distilled from Moge2 achieves the highest reconstruction quality, while DPT performs significantly worse, and Metrics3D and VGGT yield slightly lower results. This gap demonstrates that the intrinsic errors and generalization ability of the teacher model critically influence the pseudo label quality and student performance.

Effect of pseudo label quantity. We further conducted an ablation study with different numbers of pseudo label: 120, 180, 240, 300, and 360. As presented in Table 5, the results exhibit a varied trend: performance first improves as the number of pseudo labels increases, reaching its highest PSNR and SSIM at 180 samples, then slightly declines as more pseudo labels are added. With fewer pseudo labels, the student model is trained on a compact set of relatively clean labels, which reduces the influence of outliers and large teacher model errors. However, as the pseudo labels increase, the datasets become more diverse and cover a broader range of geometric structures, which theoretically enhances the generalization ability. At the same time, this results in an increase in training time and memory usage. Overall, these results suggest that the tradeoff between label representativeness and noise accumulation plays a key role in distillation performance. In our experiments, using around 240 samples achieves the best balance between supervision diversity, label reliability, and training efficiency.

Ablation study on the number of Fibonacci sampling points. To analyze the impact of the number of Fibonacci sampling points on the results, we performed an ablation study testing different sampling point values with three metrics and reconstruction time. The results are summarized in Table 6.

From the results, 20 points provided an optimal balance between the three metrics and reconstruction time. This choice aligns with common practices in the field, where 20 points sampling is widely adopted, and we also used the traditional icosahedron method as a baseline for comparison.

Under identical settings, we compare our optimizer with the traditional baseline, as shown in Table 7. The adaptive optimization achieves higher fidelity in nearly the same runtime, reducing artifacts, and improves consistency without adding significant computational cost.

## 4. Conclusions

In this work, we introduce a novel and effective framework that leverages the generative power of diffusion models to eliminate the reliance on multi-view imagery. Instead, our approach employs panoramic images as intermediate inputs to achieve globally consistent scene reconstruction. At the core of our design is a Fibonacci lattice-based initialization, which generates uniformly distributed point clouds from panoramic inputs and alleviates localized overfitting during reconstruction. In addition, we propose a dense pseudo label distillation strategy, where perspective projections derived from panoramas serve as supervisory signals, allowing the student model to inherit both structural and perceptual knowledge from a pretrained teacher model. Extensive experiments demonstrate that our framework consistently outperforms existing methods across diverse evaluation metrics. Future work will focus on enhancing higher resolution reconstruction and exploring dynamic scene modeling. One of the core challenges in dynamic scenes is handling motion blur, which can obscure fine details in the scene. Addressing this issue requires adapting our approach to effectively capture and reconstruct motion across frames, which will be an essential component in the development of immersive applications.

## Figures and Tables

**Table 1:** Mapping from scene indices (Scene1–Scene8) to full scene names.

Scene1	“A luxury bathroom”	Scene2	“A bedroom”	Scene3	“A mountain landscape”
Scene4	“Hulunbuir grassland with blue sky”	Scene5	“Waves on the beach”	Scene6	“Beijing city library”
Scene7	“An indoor exhibition hall with multiple art installations, glass display cases, large posters on the wall, and spotlights”				
Scene8	“An outdoor city plaza with a large central fountain, stone benches, tiled ground, and modern street lamps surrounded by open space”				

**Table 2:** Ablation study comparing our point cloud initialization method with the latest and most common techniques. The results, averaged across all datasets, demonstrate that our method achieves the best performance.

Method	Average		
	PSNR	SSIM	LPIPS
BiFuse	23.86	0.693	0.330
Depth anything V2	31.67	0.931	0.162
VGGT	35.80	0.904	0.213
COLMAP	38.64	0.945	0.079
COLMAP (MVS)	37.93	0.958	0.074
FlowMap	34.79	0.936	0.098
Our study	42.07	0.992	0.020

**Table 3:** Quantitative comparison of reconstruction performance on real world panoramic datasets.

Model	R1			R2			R3		
	PSNR	SSIM	LPIPS	PSNR	SSIM	LPIPS	PSNR	SSIM	LPIPS
LucidDreamer	38.68	0.987	0.047	39.73	0.988	0.043	36.14	0.982	0.049
DreamScene360	31.59	0.954	0.102	29.25	0.924	0.130	35.22	0.976	0.053
Scene4U	30.24	0.962	0.086	34.84	0.982	0.067	27.84	0.913	0.159
Our study	42.97	0.993	0.018	43.58	0.994	0.019	40.80	0.991	0.019

**Table 4:** Quantitative comparison of different teacher models in the distillation framework.

Method	Average		
	PSNR	SSIM	LPIPS
DPT	32.93	0.954	0.128
Metrics3D	41.62	0.990	0.023
VGGT	41.67	0.991	0.022
Our	42.07	0.992	0.020

**Table 5:** Ablation study on the effect of pseudo label quantity.

	Average				
	PSNR	SSIM	LPIPS	Time	GPU Usage
120	42.12	0.992	0.019	7 min 13 s	3608.04 MB
180	42.37	0.993	0.017	7 min 48 s	4328.77 MB
240	42.07	0.992	0.020	8 min 21 s	5050.23 MB
300	42.03	0.992	0.019	8 min 48 s	5770.45 MB
360	41.44	0.991	0.020	9 min 10 s	6490.01 MB

**Table 6:** Comparative quantitative analysis of different point configurations in Fibonacci sampling.

	Average			
	PSNR	SSIM	LPIPS	Time
Icosahedron (20)	41.67	0.990	0.022	8 min 12 s
15	41.58	0.990	0.023	8 min 6 s
20	42.07	0.992	0.020	8 min 21 s
25	42.24	0.991	0.022	8 min 37 s

**Table 7:** Quantitative comparison between adaptive optimization and traditional 3DGS.

Method	Average			
	PSNR	SSIM	LPIPS	Time
Transitional Gaussian Optimization	41.75	0.990	0.022	8 min 25 s
Adaptive Gaussian Optimization	42.07	0.992	0.020	8 min 21 s

## Data Availability

Part of the dataset is available on request from the authors (the data are part of an ongoing project).
